# Glucagon-like peptide-1, fibroblast growth factor 21, and other endocrine responses to alcohol ingestion in women before and after metabolic surgery

**DOI:** 10.3389/fphar.2025.1575156

**Published:** 2025-05-22

**Authors:** Mariel Molina-Castro, Blair Rowitz, Marta Yanina Pepino

**Affiliations:** ^1^ Department of Food Science and Human Nutrition, University of Illinois at Urbana-Champaign, Champaign, IL, United States; ^2^ Division of Nutritional Sciences, University of Illinois at Urbana-Champaign, Champaign, IL, United States; ^3^ Carle Illinois College of Medicine, University of Illinois at Urbana-Champaign, Champaign, IL, United States; ^4^ Carle Foundation Hospital, Department of Surgical Services & Bariatric Surgery, Urbana, IL, United States

**Keywords:** ethanol, bariatric (weight-loss) surgery, gut peptides, hypoglycemia, insulin, pharmacokinetics

## Abstract

**Indroduction:**

Glucagon-like peptide-1 (GLP-1) is integral to glucose homeostasis, appetite, and reward pathways in the brain, making GLP-1 receptor agonists effective treatments for type 2 diabetes, obesity, and potentially alcohol use disorder (AUD). Although metabolic surgery increases endogenous GLP-1, it is paradoxically associated with a higher risk of AUD.

**Methods:**

Building on cross-sectional research indicating that alcohol consumption decreases endogenous GLP-1 and may contribute to a heightened risk of hypoglycemia post-metabolic surgery, this longitudinal, within-subject study examined whether acute alcohol intake reduced GLP-1 and increased fibroblast growth factor 21 (FGF21)–a liver-derived hormone implicated in glucose regulation and alcohol consumption in animal models –more profoundly after surgery. Seven women were assessed using a randomized, crossover design; they consumed after overnight fast a standardized alcohol-containing beverage or placebo during two separate visits before surgery and repeated these interventions ∼5 months post-surgery. Blood samples were collected over 3 hours to measure blood alcohol concentration (BAC), and plasma glucose, GLP-1, FGF21, insulin, and C-peptide.

**Results:**

Post-surgery, BAC peaked faster and at higher concentrations, and alcohol clearance decreased by ∼28%–likely reflecting the loss of fat-free mass. However, the acute GLP-1 decrease and profound FGF21 increase following alcohol intake were not magnified in the postoperative period, nor did alcohol-induced reductions in glucose become more pronounced.

**Discussion:**

These findings suggest that, despite substantial weight loss and improvements in insulin sensitivity, acute alcohol consumption in the fasted state elicits comparable effects on GLP-1, FGF21, and glycemia before and a few months after metabolic surgery. Further studies with larger and more diverse cohorts are warranted to confirm these observations, clarify long-term effects on alcohol metabolism and glycemic control, and inform strategies to mitigate the potential risk of AUD in this population.

**Trial Registration:**

NCT02766322.

## 1 Introduction

Glucagon-like peptide 1 (GLP-1) is an anorectic hormone and neuropeptide produced by preproglucagon-expressing cells and neurons primarily located in the intestine and the nucleus tractus solitarii (Bloemendaal et al., 2014; Kabahizi et al., 2022) GLP-1 is secreted in response to food intake and plays critical roles in glucose homeostasis by stimulating glucose-dependent insulin secretion, inhibiting glucagon release, and slowing gastric emptying (Drucker and Nauck, 2006). Due to its role in regulating glucose levels, appetite, and food intake, GLP-1 receptor agonists (GLP-1 RAs) were initially developed to treat type 2 diabetes. However, their remarkable weight-loss effects have positioned them as the most effective pharmacological interventions for obesity (Kabahizi et al., 2022; Nuffer and Trujillo, 2015).

In addition to their efficacy in treating diabetes and obesity, GLP-1 RAs have shown potential in reducing alcohol consumption. Preclinical studies demonstrated that GLP-1 RAs decrease alcohol intake and alcohol-seeking behaviors (Marty et al., 2020; Aranäs et al., 2023; Brunchmann, Thomsen, and Fink-Jensen, 2019; Chuong et al., 2023; Vallöf, Kalafateli, and Jerlhag, 2020). While clinical trials are scarce (Klausen et al., 2022; Hendershot et al., 2025), recent findings from Hendershot et al., support the observation from several retrospective and prospective studies that GLP-1 RAs have the potential to aid in the treatment of alcohol use disorder (AUD) (reviewed in Subhani et al., 2024). Consequently, GLP-1 agonists hold promise for treating both obesity and AUD concurrently.

Alcohol consumption, however, can impair glucose control, underscoring the need to understand its impact on endogenous GLP-1 and other hormones involved in glycemic regulation. Patients who underwent metabolic surgery, such as Roux-en-Y-gastric bypass (RYGB) and sleeve gastrectomy (SG), represent a particularly relevant clinical group in this context. While these surgeries are highly effective long-term treatments for severe obesity and type 2 diabetes (Angrisani et al., 2018), evidence suggests that individuals who undergo such procedures face an increased risk of developing AUD (King et al., 2012; 2017; Ibrahim et al., 2019; Maciejewski et al., 2020) despite the significant metabolic changes induced by surgery, including elevated endogenous GLP-1 levels (Larraufie et al., 2019).

In a recent cross-sectional study, we found that acute alcohol consumption reduces endogenous GLP-1 concentrations in women who drink moderately (Molina-Castro et al., 2024). Moreover, there were no significant differences in this alcohol-related decrease in GLP-1 between women with or without a history of metabolic surgery (Molina-Castro et al., 2024). We also found that, despite decreasing plasma GLP-1 concentrations, 28% of women assessed post-surgery experienced plasma glucose in the hypoglycemic range, contrasting with none in the non-surgery control group (Molina-Castro et al., 2024). The reason underlying the increased risk for alcohol-induced hypoglycemia post-metabolic surgery is unclear, but we hypothesize it may be related, at least in part, to alcohol’s greater inducement of fibroblast growth factor 21 (FGF21) after surgery.

FGF21 is a liver-derived hormone that lowers blood glucose and reduces alcohol and sugar intake in animal models (Jensen-Cody et al., 2020; Flippo et al., 2022), and its secretion is enhanced within the first few months following metabolic surgery (Harris et al., 2017; Luca et al., 2023). Here, using secondary data from a prospective study, we evaluated the same women before and after metabolic surgery to determine whether acute alcohol consumption further lowers endogenous GLP-1 post-surgery and test the hypothesis that alcohol-induced plasma FGF21 and the reduction in plasma glucose are more pronounced after, rather than before surgery.

## 2 Materials and methods

### 2.1 Participants

The women enrolled in this study were included in a parent study with the primary aim of understanding the effects of different bariatric surgeries on alcohol pharmacokinetics and pharmacodynamics (Clinical trial registration NCT02766322). The parent study included a longitudinal arm: women who were planning to undergo bariatric surgery—assessed before and when reaching ∼16% body weight loss, typically within the first year after undergoing surgery, and a cross-sectional arm: women who underwent bariatric surgery within the last 5 years, and a non-operated control group. The parent study focused on women exclusively because less than 20% of patients undergoing these surgeries are men (Fuchs et al., 2015), and there are sex-related differences in alcohol pharmacokinetics (Baraona et al., 2001). During screening, women were excluded if they were alcohol abstainers, were drinking more than seven standard drinks per week, or were drinking more than four per drinking occasion (one standard drink contains 14 g of pure ethanol). They were also excluded if they were pregnant or breastfeeding, younger than 21 years of age or older than 64, or had anemia, malabsorptive diseases, inflammatory diseases, liver or kidney disease or severe organ dysfunction, cancer within the last 5 years, a diagnosis of alcohol abuse or dependence (based on an interview with the Semi-Structured Assessment for Genetics of Alcoholism (SSAGA) that refers to DSM-IV diagnostic terminology) or current regular use of drugs with potential for misuse. Also exclusionary were taking medications that could affect alcohol metabolism, being currently smoking cigarettes or having quit less than 2 months ago, or having a body weight >450 pounds (because of a weight limit on the dual-energy X-ray absorptiometry (DXA) used to assess body composition for calculating alcohol dose). A total of 82 women underwent screening for eligibility in the parent study, of which 14 were recruited for the longitudinal arm of the study. Unfortunately, the study was challenged by the COVID-19 pandemic, and only seven of these 14 women completed pre- and post-assessments, which are included in this manuscript. Data on alcohol pharmacokinetics from participants in the parent study have been reported previously (Acevedo et al., 2020), and cross-sectional data on plasma GLP-1 and glucose concentrations from four women (post-surgery values only) were included in a recent publication (Molina-Castro et al., 2024). All research procedures were approved by the Institutional Review Boards of the University of Illinois at Urbana-Champaign (UIUC) and the Carle Foundation Hospital (CFH), guidelines from the National Institute on Alcohol Abuse and Alcoholism (NIAAA) on “Administering Alcohol in Human Studies” were followed, and women provided written informed consent before participating in the study.

### 2.2 Study design and experimental procedures

The study was conducted in a private room in CFH or the Clinical Research Suite at (UIUC). To assess inclusion/exclusion criteria, women took part in a screening visit, and when eligible, they were invited to participate in the study. Using a randomized crossover design, they were evaluated before surgery in two “oral alcohol challenge sessions” (one alcohol and one placebo), approximately 1 week apart. The sessions were repeated after surgery when they lost approximately 20% of their body weight. Participants consumed either 0.5 g of alcohol per kg of FFM (equivalent to ∼ two standard alcoholic beverages: alcohol condition) or a non-alcohol placebo beverage (control condition) on visit one and the other beverage on visit two. The dose of alcohol consumed was based on each participant’s total fat-free mass (FFM), because FFM, not body weight, correlates closely with alcohol volume of distribution (Gentry, 2000). A human chorionic gonadotropin urine test was performed at the start of each study session (including the screening visit) to exclude women who were pregnant.

#### 2.2.1 Screening visit

After obtaining informed consent, participants were screened with a detailed history, including a review of pre-surgical medical records. Routine blood tests were performed to screen for liver and other potential organ disease and anemia. The SSAGA was used to identify cases of alcohol dependence and alcohol and drug use history. We determined FFM using the Lunar iDXAtm (GE Healthcare).

#### 2.2.2 Oral alcohol challenge sessions (alcohol and placebo beverages)

On all testing days, participants arrived at approximately 0800 after an overnight fast at home. After checking vital signs, an intravenous line attached to a three-way stopcock was inserted into a superficial dorsal hand vein. The hand was heated in a hotbox (50°C) to obtain arterialized venous blood, and the catheter was kept patent by slow saline infusion. At approximately 0930 h, participants consumed a standard dose of alcohol (0.5 g/kg FFM) or a nonalcoholic placebo control beverage. Alcohol beverages were prepared with 190-proof ethanol at 20% vol/vol mixed with a noncaloric flavored drink (Kool-Aid, Kraft Heinz Company, Chicago, IL) sweetened with Splenda (Heartland Consumer Products, Carmel, IN). Placebo beverages were prepared with only the noncaloric flavored drink sweetened with Splenda. The final volume for alcohol and placebo beverages was kept the same. The beverages were served in two cups containing half a dose each, consumed within two consecutive 5-min periods (for a total of 10 min). During both conditions (i.e., alcohol and placebo), 2 mL of ethanol was sprayed onto the surface of each cup to serve as a smell and flavor mask (Acevedo et al., 2020). Blood samples were taken just before and 5, 10, 15, 20, 25, 35, 45, 60, 75, 90, 105, 120, 135, 150, 180, and 210 min after drinking the beverage. An aliquot of blood was immediately processed to measure blood alcohol concentrations (BAC) using gas chromatography as described previously (Acevedo et al., 2020). The remaining blood sample was collected in chilled EDTA tubes containing a protease inhibitor cocktail. These samples were placed on ice and centrifuged at 4°C, and the plasma was stored at −80°C for subsequent analyses of glucose, hormones, and gut peptides.

#### 2.2.3 Biochemical measurements

Plasma glucose concentrations were measured at times 0, 15, 20, 25, 35, 45, 60,90, 120, 150, and 180 min using a biochemistry analyzer (YSI 2300 STAT plus; Yellow Spring Instrument Co., Yellow Springs, OH). Plasma active GLP-1 (here referred to as GLP-1) was extracted with 95% ethanol, dried under nitrogen, rehydrated with sample hydrating solution, and then assayed by Radioimmunoassay per kit instructions (Millipore RIA kits, Billerica MA). To make a more efficient use of the kits, we included the same time points as for glucose, except that we excluded timepoint 150 min. Insulin and C-peptide were measured at the same time points as GLP-1 by electrochemiluminescence using Roche Elecsys kits on the Roche Cobas e 601 module for immunoassay tests (Roche Diagnostics, Indianapolis, IN). GLP-1, insulin, and C-peptide were measured at the Core laboratory at Washington University in St. Louis. Plasma FGF21 concentrations were determined in-house using an enzyme-linked immunosorbent assay kit (R&D Systems, Minneapolis, MN). The following time points were included: 0, 35, 75, 105, 135, 180, and 210 min. We pilot-tested plasma concentrations of FGF21 on the placebo day and found that there were no significant differences across time. Therefore, to be able to run in duplicate all plasma samples from a participant (for both conditions, before and after surgery) within the same kit, we excluded the timepoint 35 min on the placebo condition and replaced that value with the average of the previous and subsequent sample. The total areas under the curve versus time (AUC) for glucose, all the hormones, and alcohol were calculated using the trapezoid method as previously described (Allison et al., 1995).

#### 2.2.4 Classical alcohol pharmacokinetic measures

We determined peak BAC, time-to-peak BAC, pre- and post-surgery for each participant. We also determined the disappearance rate of alcohol (β_60_), the total amount of alcohol eliminated per hour (b_60_), and the alcohol elimination rate (R) as previously described (Pepino, Steinmeyer, and Mennella, 2007). The disappearance rate of alcohol (β_60_) was estimated for each participant from the slope of the linear least-squares regression lines within the apparent linear portion of the descending limb of the BAC vs time curve. To exclude the upper distribution phase and lower first-order elimination phase of the apparent linear portion of the curve, the first value was taken 0.5 h after the peak BAC, and all subsequent readings ≥0.20 g/L were used. The total amount of alcohol eliminated per hour (b_60_) was calculated as follows: b_60_ = β_60_ × TBW/B_w_, with Total Body Water (TBW), TBW = [0.1069 × height (cm)] + [0.2466 × weight (kg)] – 2.097, and B_w_ = 0.80. This standardized anthropometric equation estimates TBW for women with a precision of ±9%–11% (Watson, Watson, and Batt, 1980). Alcohol elimination rate (R) was expressed as the amount of alcohol eliminated per kilogram of the body per hour (R = b_60_/weight).

### 2.3 Statistical analysis

To determine whether acute alcohol consumption affected plasma glucose, GLP-1, insulin, C-peptide, or FGF21 differently before and after metabolic surgery, we conducted repeated measured variance analyses that included three within-subject factors. That is, “Time” (before and after surgery), “Condition” (placebo and alcohol), and Trials (1–11 for glucose, 1–10 for GLP-1, insulin and C-peptide, or one to seven for FGF21). For plasma sample concentrations that were below the assay’s sensitivity limit of detection, we used a value equivalent to half of the limit of detection. When applicable, significant interactions were further analyzed using Fisher’s least significance difference tests. Statistical significance was set at 2-sided P < 0.05. When the sphericity assumption was violated, we used adjusted P-values using the Huynh-Feldt and Greenhouse-Geisser corrections. We performed all analyses in Statistica version 14.0.1.25 (TIBCO Software Inc.) and created graphs using GraphPad Prism version 10.4.1 for Windows, GraphPad Software, Boston, Massachusetts, United States.

## 3 Results

### 3.1 Participant characteristics

The study cohort characteristics are shown in Table 1. Seven women (3 RYGB and 4 SG) completed the study. They were, on average, 45.5 (SD = 13.4) years old, and all were Caucasian and non-Hispanic. Three had a diagnosis of diabetes before surgery and were treated with insulin and/or metformin, but none were treated with GLP-1 agonists. As expected, body weight, FFM, fasting plasma insulin, C-peptide, and the Homeostatic Model Assessment for Insulin Resistance (HOMA-IR2) were lower after than before surgery (Table 1).

**TABLE 1:** Characteristics of study participants and alcohol-related variables.

Characteristic	Before surgery	After surgery	*P* Value
Weight, kg	117.2 (11.7)	95.6 (11.1)	0.000
BMI, kg/m^2^	44.9 (3.9)	36.7 (3.6)	0.000
FFM, kg	55.0 (3.6)	50.0 (5.2)	0.02
Time from surgery, y	NA	0.38 (0.26)	NA
Fasting plasma concentrations			
Glucose, mg/dL	120.6 (42.7)	91.0 (11.5)	0.06
Insulin, µIU/mL	26.0 (10.6)	9.8 (6.2)	0.001
C-peptide, ng/mL	4.2 (1.8)	2.8 (1.4)	0.002
Active GLP-1, pmol/L	21.0 (8.6)	11.3 (5.1)	0.06
FGF21, pg/mL	311.8 (135.9)	694.3 (950.4)	0.31
HOMA-IR2	3.5 (1.2)	1.4 (0.8)	0.001
Alcohol-related variables			
Age			
Onset of alcohol drinking, y	15.1 (4.7)		
Regular drinking began, y	22.4 (4.3)		
Drinking over the past 6 months			
No. of drinking days per month^a^	5.1 (3.7)	1.7 (1.8)	0.054
No. of alcoholic drinks per drinking day	1.9 (1.0)	1.3 (0.8)	0.22
Classical alcohol pharmacokinetics			
Peak BAC, g/L	67.6 (16.5)	106.7 (24.2)	0.01
Time to reach peak BAC, h^b^	0.5 (0.1)	0.3 (0.1)	0.02
Area under the BAC time curve, g/L × h_0-3_	90.9 (17.4)	128.8 (16.5)	0.001
Alcohol elimination measures			
Disappearance rate, β _60_, g/L/h	0.21 (0.05)	0.17 (0.04)	0.12
Total eliminated, b_60_, g/h	11.5 (3.3)	8.3 (2.0)	0.03
Elimination rate, R, g/h/kg	0.10 (0.03)	0.09 (0.02)	0.33
No. of standard drinks given on alcohol challenge test	2.0 (0.1)	1.8 (0.2)	0.02

Data are presented as mean (SD).

Abbreviations: BAC, blood alcohol concentration; BMI, body mass index (calculated as weight in kilograms divided by height in square meters); FFM, fat-free mass; NA, not applicable.

^a^ Due to incomplete answers in one of the SSAGA, interviews after surgery, this variable includes only six participants.

^b^ From the time of the first sip of alcoholic beverage, consumed over 10 min.

### 3.2 Effect of metabolic surgery on alcohol pharmacokinetics

**FIGURE 1:**
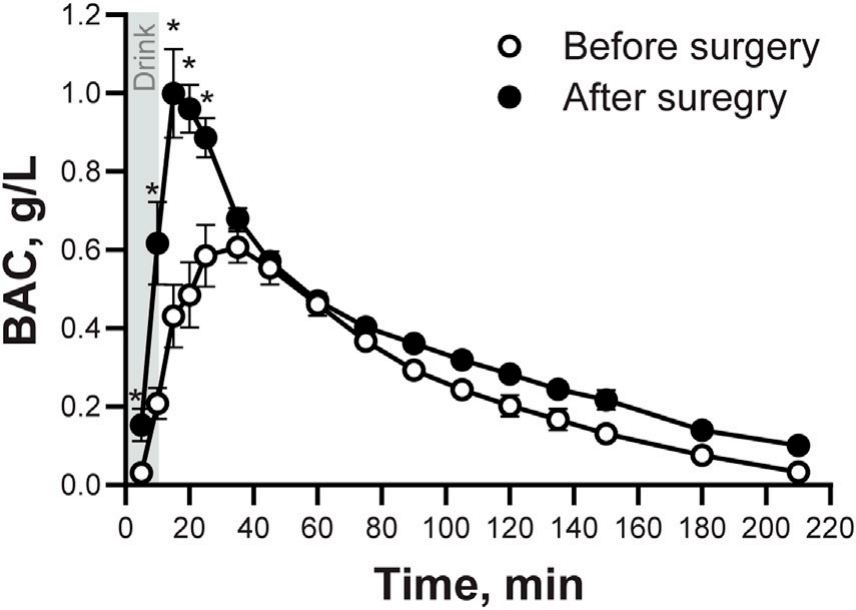
Blood Alcohol Concentrations (BAC) following the ingestion of an alcohol drink, before and after undergoing metabolic surgery. Blood alcohol concentrations (BAC) in seven women after ingestion of an alcohol drink (0.5 g/kg fat-free mass) before and ∼5 months after surgery. BAC data was analyzed using repeated measures ANOVA analysis. Trials and Time (before and after surgery), as well as Time × Trial interaction, were included in the model. Significant effects: Time, *F*_(1,6)_ = 42.92, *P* = 0.0006; Trials, *F*_(15,90)_ = 56.31, *P* < 0.0001; Time x Trials, *F*_(15,90)_ = 11.27, *P* < 0.0001. Data are presented as mean values ± SEM. *Signifies different than before surgery for at that same time point. Significance was set at P < 0.05.

In agreement with results from the cross-sectional arm of the parent study, peak BAC was higher, time-to-peak BAC happened sooner, and AUC was bigger after than before surgery (Figure 1; Table 1). There were no differences between before and after surgery in alcohol’s disappearance rate ( β _60_) or the elimination rate (R), but the total amount of alcohol eliminated per hour (b_60_) was smaller after than before surgery (Table 1).

### 3.3 Effects of metabolic surgery and alcohol consumption on plasma glucose, insulin, and C-peptide concentrations

**FIGURE 2:**
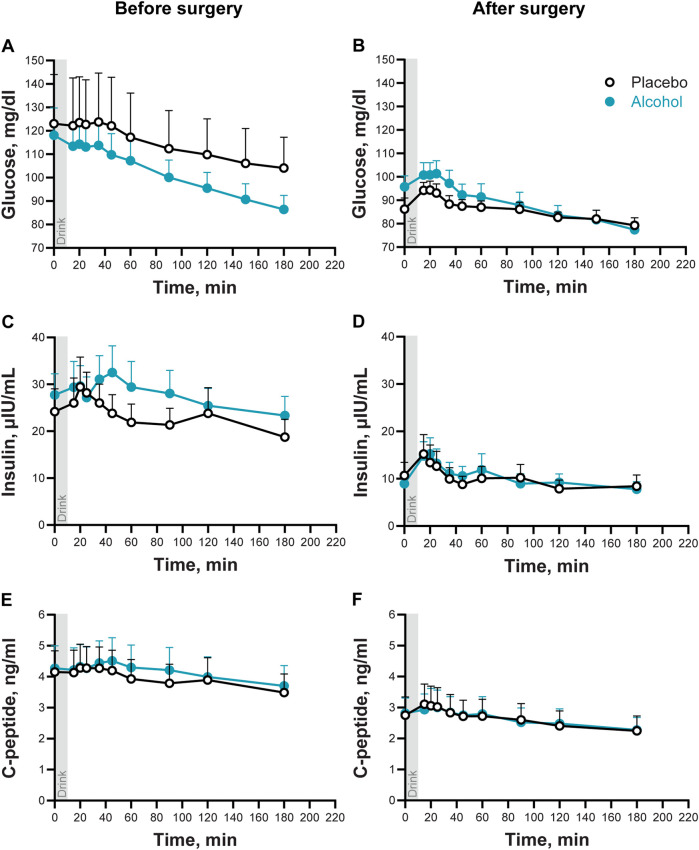
Plasma glucose, insulin, and C-peptide concentrations following the ingestion of placebo and alcohol drinks, before and after metabolic surgery. Plasma glucose **(A, B)**, insulin **(C, D)**, and C-peptide **(E, F)** concentrations in seven women following ingestion of an alcohol drink (0.5 g/kg fat-free mass; teal symbols) or placebo (non-alcohol version sprayed with 2 mL of alcohol; black symbols) over 10 min (gray bar). Data are shown for before and after metabolic surgery (left and right panels, respectively). Glucose, insulin, and C-peptide concentrations were analyzed using repeated measures ANOVA analyses. Condition (placebo and alcohol), Trials, and Time (before and after surgery), as well as all interactions, were included in the model. Significant effects for glucose: Time x Trials, *F*_10,60_ = 3.30, *P* < 0.002; Adj P = 0.08; Condition x Trials, *F*_10,60_ = 6.88, *P* < 0.0001; Adj P < 0.008. Significant effects for insulin: Time, *F*_1,6_ = 44.87, *P* = 0.0005; Trials, *F*_9,54_ = 7.96, *P* < 0.0001; Adj P < 0.002. Significant effects for C-peptide: Time, *F*_1,6_ = 35.50, *P* = 0.001; Trials, *F*_9,54_ = 19.07, *P* < 0.0001; Adj P < 0.002. Data are presented as mean values with SEM shown as upper error bars.

Plasma glucose concentrations tended to be lower, and glucose AUC tended to be smaller, overall after than before surgery (Time × Trial: F_10,60_ = 3.30; P < 0.002; Adj P = 0.08; Figure 2; Table 2), and glucose excursions over time differed between the two conditions (Condition × Trial: F_10,60_ = 6.88; P < 0.0001; Adj P < 0.008; Figure 2). Compared to baseline, plasma glucose concentrations decreased by the end of the visit in both conditions, but the decrease was greater after alcohol than placebo drinks. Alcohol lowered blood glucose concentrations to the hypoglycemic range in two women (28%) after surgery vs none before surgery. There was also one woman who only experienced plasma glucose in the hypoglycemic range after surgery during the placebo condition. None of the participants required treatment for hypoglycemia. There was no interaction between Time × Condition × Trial for plasma glucose (P > 0.98).

**TABLE 2:** Areas under the curve.

Variable	Before surgery		After surgery		P-values		
	Placebo	Alcohol	Placebo	Alcohol	Time	Cond	Time x Cond
AUC Glucose, mg/dL × h_0-3_	341.6 ± 51.3	304.1 ± 22.2	256.9 ± 8.4	265.6 ± 13.8	0.07	0.31	0.35
AUC Insulin, µIU/mL × h_0-3_	68.7 ± 12.6	82.0 ± 13.1	29.1 ± 7.1	30.3 ± 6.3	0.0006	0.28	0.34
AUC C-peptide, ng/mL × h_0-3_	11.7 ± 2.0	12.4 ± 2.1	7.8 ± 1.6	7.8 ± 1.4	0.001	0.58	0.61
AUC GLP-1, pmol/L × h_0-3_	74.1 ± 24.1	39.4 ± 14.8	40.6 ± 4.5	24.2 ± 3.9	0.25	0.0004	0.27
AUC FGF21 x10^3^, pg/mL × h_0-3_	67.2 ± 14.0	262.8 ± 73.4	146.8 ± 73.9	321.3 ± 122.0	0.44	0.02	0.68

Data are presented as mean ± SEM.

Plasma insulin and C-peptide concentrations were overall lower, and insulin and C-peptide AUCs were smaller after surgery than before surgery (Main effect of Time for insulin: F_1,6_ = 44.9; and for C-peptide F_1,6_ = 35.4; both P ≤ 0.001; Figures 2B, C and Table 2). Compared to baseline concentrations, ingestion of a drink (placebo or alcohol) was associated with a transient and subtle increase of plasma insulin and C-peptide (Main effect of Trial for insulin: F_9,54_ = 8.0 and for C-peptide: F_9,54_ = 19.1, both P < 0.001; Adj P < 0.002). There were no interactions between any factors for plasma insulin or C-peptide.

### 3.4 Effects of metabolic surgery and alcohol consumption on plasma GLP-1 concentrations

**FIGURE 3:**
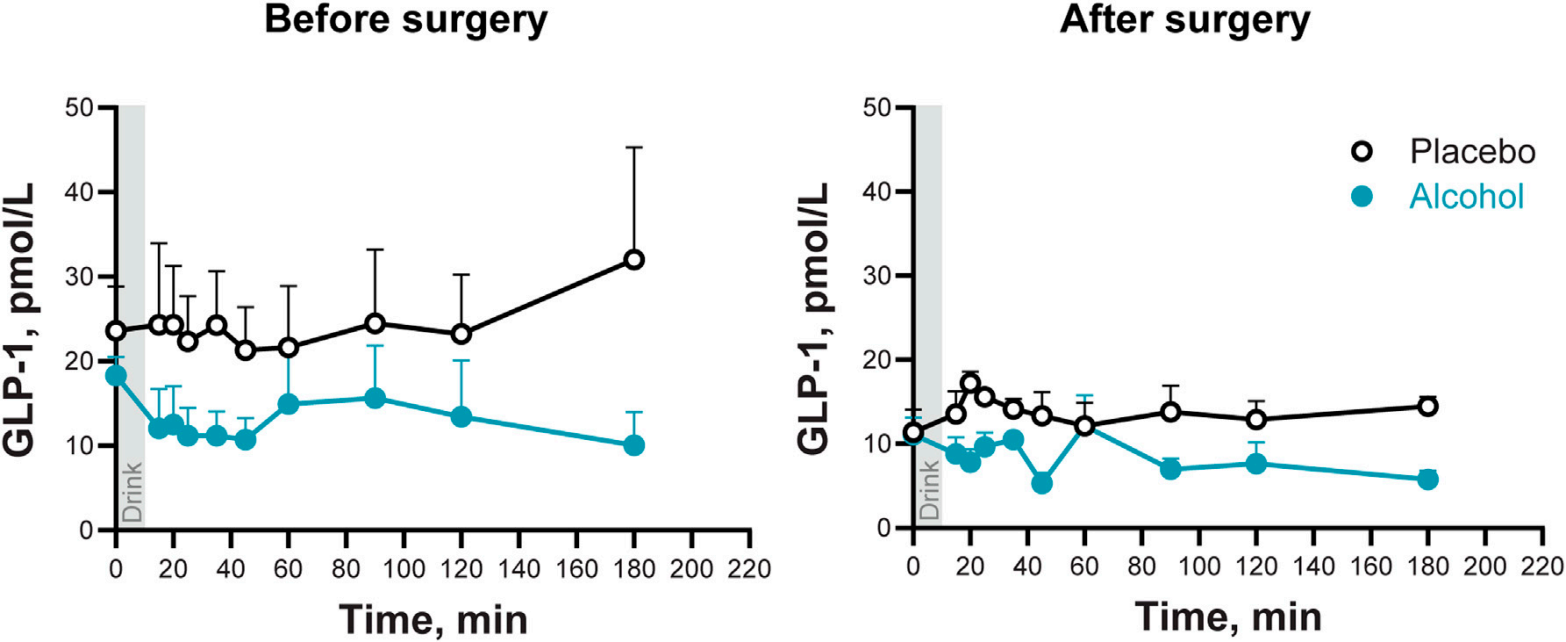
Plasma GLP-1 concentrations following the ingestion of placebo and alcohol drinks, before and after undergoing metabolic surgery. Plasma GLP-1 concentrations in seven women after ingestion of an alcohol drink (0.5 g/kg fat-free mass; teal symbols) or placebo (black symbols) over 10 min (gray bar). Data are shown for before and after metabolic surgery (left and right panels, respectively). GLP-1 concentrations were analyzed using repeated measures ANOVA analyses. Condition (placebo and alcohol), Trials, and Time (before and after surgery), as well as all interactions, were included in the model. Significant effects: Condition, *F*_1,6_ = 3.34, *P* = 0.001. Data are presented as mean values with SEM shown as upper error bars.

On the day of alcohol consumption, women had lower plasma GLP-1 concentrations and smaller GLP-1 AUC than on the placebo day, both before and after surgery (Main effect of Condition: F_1,6_ = 30.4; P < 0.002; Figure 3; Table 2). There was no main effect of Time (before surgery and after) nor other significant interactions (all P values >0.24).

### 3.5 Effects of metabolic surgery and alcohol consumption on plasma FGF21 concentrations

**FIGURE 4:**
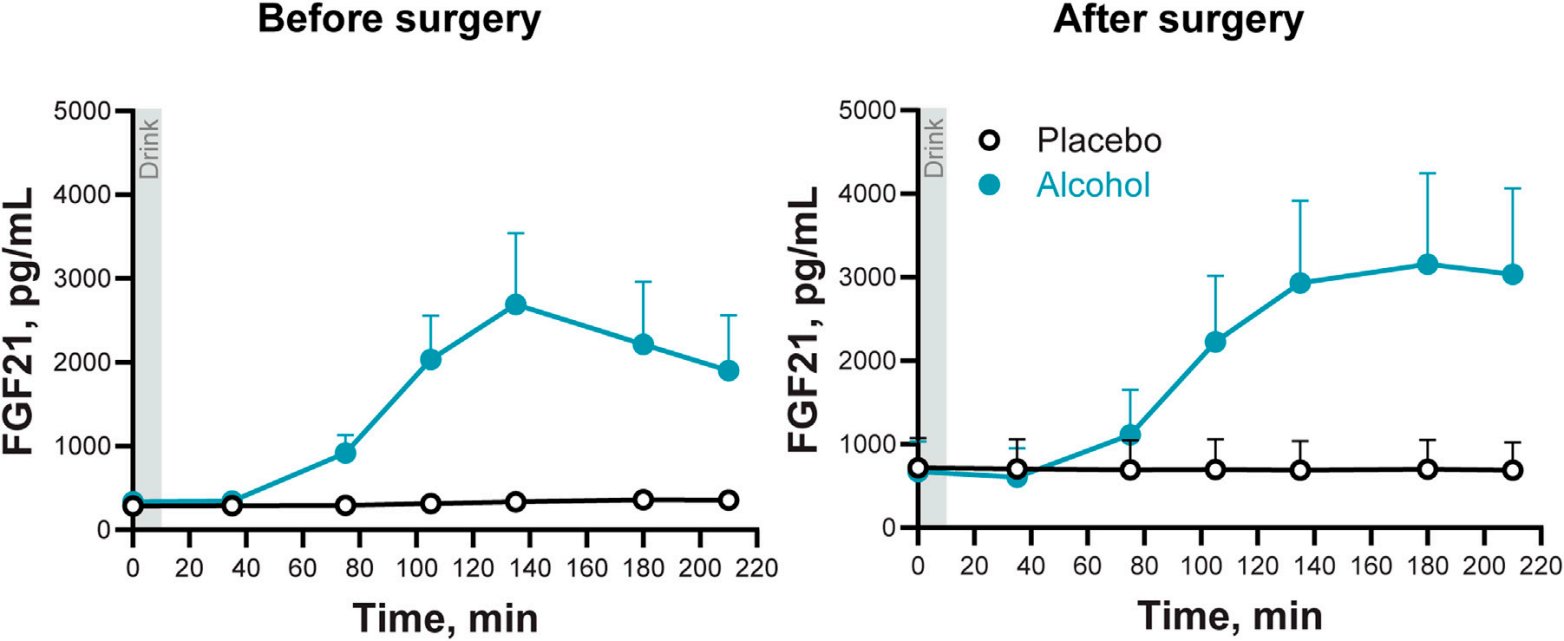
Plasma FGF21 concentrations following the ingestion of placebo and alcohol drinks, before and after undergoing metabolic surgery. Plasma FGF21 concentrations in seven women after ingestion of an alcohol drink (0.5 g/kg fat-free mass; teal symbols) or placebo (black symbols) over 10 min (gray bar). Data are shown for before and after metabolic surgery (left and right panels, respectively). FGF21 concentrations were analyzed using repeated measures ANOVA analyses. Condition (placebo and alcohol), Trials, and Time (before and after surgery), as well as all interactions, were included in the model. Significant interactions: Condition x Trials, *F*_6,36_ = 9.82, *P* < 0.0001; Adj P = 0.018; Time x Condition x Trials, *F*_6,36_ = 2.45, *P* = 0.04; Adj P = 0.15. Data are presented as mean values with SEM shown as upper error bars.

Alcohol ingestion acutely stimulated circulating FGF21 (Main effect of Condition: F_1,6_ = 10.3; P = 0.018), tripling the FGF21 AUC both before and after surgery (F_1,6_ = 11.1; P = 0.016). The highest post-alcohol concentration of FGF21 was measured at 135 min post-consumption (Condition × Trial: F_6,36_ = 9.8; Adj P = 0.018; Figure 4). Although there was a trend for the alcohol-induced increase in FGF21 to persist longer after surgery than before surgery, the significant value of the interaction did not survive the adjustment for sphericity violation (Time × Condition × Trial: F_6,36_ = 2.45; P = 0.04; Adj P = 0.15; Figure 4).

## 4 Discussion

Results from this longitudinal study confirm and extend previous findings regarding how metabolic surgery affects alcohol pharmacokinetics and, in turn, how acute alcohol consumption influences plasma glucose and glucoregulatory hormones after surgery. Consistent with earlier research that included the same women before and after surgery (Pepino et al., 2015; Engel et al., 2022); or different control groups, including non-surgical controls (Pepino et al., 2015; Acevedo et al., 2018; Seyedsadjadi et al., 2022), BAC peaked faster and reached higher concentrations following surgery. However, alcohol intake decreased plasma endogenous GLP-1 concentrations to a similar degree both before and after surgery. In line with findings from studies in rodents (Liu et al., 2016; Song et al., 2018) and humans (Søberg et al., 2018; Desai et al., 2017; Lanng et al., 2019; Farokhnia et al., 2023), alcohol robustly and acutely increased FGF21 plasma concentrations; yet, contrary to our hypothesis, neither alcohol-induced changes in plasma FGF21 nor changes in plasma glucose differed between the pre-and post-surgery assessments in the same participants.

Previous research on alcohol’s acute effects on endogenous plasma GLP-1 had yielded mixed findings. Some studies indicate that alcohol exposure—whether oral (Farokhnia et al., 2022; Molina-Castro et al., 2024) or intravenous (Farokhnia et al., 2022)—reduces endogenous GLP-1 response, while others report no effect (Calissendorff et al., 2012; Lanng et al., 2019). The findings from this longitudinal study, consistent with our earlier cross-sectional results, including women assessed on average 1.5 years post-metabolic surgery (Molina-Castro et al., 2024), suggest that alcohol’s acute effects on GLP-1 are subtle. Specifically, a decrease in plasma GLP-1 for a few hours following beverage consumption becomes apparent only when comparing GLP-1 concentrations on the alcohol day to those on the placebo day. Whether this decrease in an anorectic hormone contributes to alcohol’s known “apéritif effect” —i.e., increasing food intake (Eiler et al., 2015; Yeomans 2004)— remains unclear, and future studies could explore this possibility.

Contrary to our hypothesis, the alcohol-related decrease in plasma glucose was not more pronounced after than before surgery. Instead, after surgery, oral consumption of the beverage caused a transient increase in plasma glucose—an effect also reported in previous studies involving women assessed post-metabolic surgery (Acevedo et al., 2019; Molina-Castro et al., 2024). At first glance, these results seem to conflict with our previous cross-sectional study, which found that compared to non-surgery control women, those who had undergone metabolic surgery were at higher risk of hypoglycemia after consuming the same dose of alcohol (Molina-Castro et al., 2024). However, in that cross-sectional study, the surgery group was compared to a non-surgery control group matched in body composition and other characteristics, and diabetes was an exclusionary criterion. In the current study, despite the advantage of tracking the same women before and after surgery, there were major changes in body composition as expected, and three out of seven (43%) of participants had diabetes prior to surgery. Although their insulin sensitivity improved considerably post-surgery, they remained more insulin-resistant than the individuals in our earlier study. Rather than indicating a contradiction, these findings highlight how different comparator groups can offer distinct insights when interpreting results.

The decrease in plasma glucose observed after alcohol intake in women before surgery is expected and likely caused by alcohol’s inhibitory effects on glucose production (Siler et al., 1998). However, the mechanism underlying the transient rise in plasma glucose after alcohol consumption in women assessed post-surgery—though consistent with previous studies (Acevedo et al., 2019; Molina-Castro et al., 2024)—remains unclear. As previously proposed (Molina-Castro et al., 2024), this temporary increase may reflect the acute impact of rapid alcohol intoxication on liver metabolism. Supporting this hypothesis, a clinical study in which non-operated participants reached a comparable peak BAC within 15 min from an intragastric alcohol bolus administration showed a similar plasma glucose response—an initial rise of about 7 mg/dL above baseline, followed by a gradual decline (Lanng et al., 2019).

In line with previous findings (Acevedo et al., 2018), we observed that the total amount of alcohol cleared per hour (b60) decreased by about 28% after surgery compared to before surgery. Recent studies using intravenous alcohol clamping—a method that precisely measures the alcohol elimination rate (AER)—have shown that FFM, a strong predictor of lean liver volume (Sinha et al., 2020), accounts for most of the variance in AER in women (Seyedsadjadi et al., 2023; Vatsalya et al., 2023). Accordingly, total alcohol clearance per hour would be expected to decrease when participants lose an average of 21.6 kg, 23% of which was FFM. Indeed, once adjusted for body weight (R), alcohol elimination measures no longer differed significantly between the pre- and post-surgery assessments.

Here, we also demonstrate that, consistent with previous clinical studies (Søberg et al., 2018; Desai et al., 2017; Lanng et al., 2019; Farokhnia et al., 2023), alcohol acutely and substantially increases plasma FGF21 concentrations. Our findings extend prior results observed in healthy, normal-weight individuals (Søberg et al., 2018; Lanng et al., 2019; Desai et al., 2017) and in non-treatment-seeking AUD participants (Farokhnia et al., 2023)—mostly male—to women with severe obesity assessed before and after metabolic surgery. Although findings from previous studies on metabolic surgery’s effects on FGF21 are conflicting, they overall suggest that plasma FGF21 concentrations rise during the first few months after surgery and return to pre-operative concentrations by the end of the first postoperative year (Luca et al., 2023; Hosseinzadeh, Roever, and Alizadeh, 2020). Accordingly, we found a non-significant increase in fasting plasma FGF21 concentrations after surgery, and we found that alcohol ingestion elicits similar robust increases in FGF21 both before and after surgery.

These findings should be interpreted in light of the study’s strengths and limitations. To our knowledge, this is the first investigation examining how alcohol ingestion influences GLP-1 and FGF21 plasma concentrations in the same participants before and after metabolic surgery. Unlike previous work on the effects of alcohol ingestion on FGF21, this study employed a crossover, placebo-control design, minimizing inter-subject variability and allowing us to distinguish alcohol-specific effects from natural endocrine fluctuations over time that can take place in the fasted state. Because most patients undergoing metabolic surgery are women (Fuchs et al., 2015), men were not included—an acknowledged limitation. Although the sample size (n = 7) is small, the prospective study design, coupled with a crossover, placebo-control condition, confers high power and statistical efficiency. In addition, we could not verify participants’ adherence to the overnight fasting requirement at home, which could confound GLP-1 findings. While we usually rely on fasting plasma glucose and insulin concentrations to confirm compliance in studies excluding individuals with diabetes, this approach is not feasible when including people with diabetes. Nevertheless, the similarity of results between this longitudinal study and the cross-sectional study (which excluded diabetes) suggests that diabetes likely did not influence findings on the effects of alcohol on GLP-1. In conclusion, our findings indicate that although metabolic surgery substantially alters the pharmacokinetics of alcohol—leading to a more rapid and higher peak BAC—the acute alcohol-induced decrease in endogenous GLP-1 and increase in FGF21 are not exacerbated in the early postoperative period. This suggests that despite considerable weight loss and improvements in insulin sensitivity, acute alcohol consumption (in the fasted state) elicits broadly similar endocrine responses before and within a few months after surgery. Future studies with larger, more diverse samples that investigate the acute effects of alcohol consumption in the fasted and the fed state are warranted to confirm these results and further clarify how surgery-related physiological changes may affect the interplay between alcohol metabolism, glucoregulatory hormones, and glycemia over the longer term.

## Data Availability

The datasets presented in this study can be found in online repositories. The names of the repository/repositories and accession number(s) can be found below: The datasets analyzed for this study can be found in the Illinois Data Bank [https://databank.illinois.edu/datasets/IDB-1059570?code&equals;_GREjZTdPDx7pF9LskLi8qw3m4i9hng3Y4bd2_XANb0].
